# Differential Epigenetic Regulation in Uninfected and Tuberculosis–Human Immunodeficiency Virus Co-Infected Patients

**DOI:** 10.3390/microorganisms12051001

**Published:** 2024-05-16

**Authors:** Katlego Mamabolo, Reubina Wadee, Yvonne Perner, Pumza Magangane, Sanelisiwe Thinasonke Duze, Musa Marimani

**Affiliations:** 1 Anatomical Pathology, School of Pathology, Health Sciences, University of the Witwatersrand, Johannesburg 2001, South Africareubina.wadee@nhls.ac.za (R.W.);; 2 Clinical Microbiology and Infectious Diseases, School of Pathology, Health Sciences, University of the Witwatersrand, Johannesburg 2001, South Africa

**Keywords:** HIV, AIDS, *Mtb*, TB, epigenetics, gene expression

## Abstract

This study aimed to compare the degree of epigenetic modifications between a TB-HIV co-infected cohort and uninfected subjects. Formalin-fixed paraffin-embedded (FFPE) tissues were retrieved from 45 TB-HIV co-infected and 45 control individuals. Real-time PCR was applied to compare the level of expression of genes involved in epigenetic regulation. The protein multiplex assay was used to assess the degree of protein modification. DNA sequencing was used to determine the evolutionary relationships between the infecting HIV and *Mtb* strains. Our results indicated a significant increase in the expression of the five candidate genes in the patients with TB-HIV relative to the control cohort. A sharp increase in the degree of histone methylation, acetylation and phosphorylation was observed in TB-HIV co-infected patients. The phylogenetic analysis classified the strains into three distinct HIV clusters and five *Mtb* clusters. The disparities in the expression profiles of our candidate genes between the TB-HIV cohort and non-TB-HIV group highlights the important role played by various TB and HIV strains in regulating the host gene expression landscape.

## 1. Introduction

Human immunodeficiency virus (HIV) is a retrovirus that attacks the cells within the immune system, predominantly the CD_4_^+^ cells, and culminates in Acquired Immunodeficiency Syndrome (AIDS) [1,2]. HIV-1 is more virulent and is responsible for the global AIDS pandemic due to its high transmissibility. This strain is classified into four separate groups based on variations in the envelope gene: the M group, O group, P group (newest group) and N group (non-M, O or new) [3,4].

The origins of *Mycobacterium tuberculosis* (*Mtb*) in terms of human infection remain elusive [5]. Tuberculosis (TB) is caused by bacteria within the Mycobacterium Tuberculosis Complex (MTBC) [6,7,8]. Overall, *Mtb* is the primary cause of death in patients infected with HIV [8,9]. Approximately 10 million people had TB in 2021 and about 300,000 HIV-positive patients died from TB-related causes [10]. Epigenetics is the study of changes in gene expression that do not alter the DNA sequence. The nucleosome contains positively charged histone proteins which have an exposed N-terminal tail that is vulnerable to posttranscriptional modifications [11]. These modifications lead to alterations in chromatin accessibility and result in the up-regulation or down-regulation of a specific gene [11]. Common histone modifications include acetylation, phosphorylation and methylation [12,13,14]. It has been shown that infection with HIV or *Mtb* can drastically change the levels of acetylation, phosphorylation and methylation. Our current study was aimed at comparing the degree of epigenetic modifications in the formalin-fixed paraffin-embedded (FFPE) human tissues collected from patients co-infected with TB-HIV and non-TB-HIV control subjects. This was achieved by assessing the expression of the *DNA methyltransferase1* (*DNMT1*), *SET domain bifurcated histone lysine methyltransferase2* (*SETDB2*) and *histone lysine methyltransferase* (*KMT*) genes which are involved in methylation, as well as the expression of the *Class II transactivator* (*CIITA*) and *histone acetyltransferase* (*HAT*) genes which are implicated in histone acetylation. These five host genes were investigated as their expression profiles have not been widely researched in patients co-infected with TB-HIV, especially in sub-Saharan Africa. We also interrogated the evolutionary relationship between the infecting HIV and *Mtb* strains. In particular, the demographic makeup, genetic variability and the TB- and HIV-drug-resistant profile in Sub-Saharan Africa is unique and different from other geographical locations. These features may be important as they may significantly affect the epigenetic processes involved leading to significant changes in gene expression, disease progression and severity. In contrast to most previous studies, our investigation utilized human lymph node FFPE biopsies as a source of *Mtb* and HIV microbial agents.

## 2. Methods

### 2.1. Ethics and Sample Collection

Initially, human biopsy samples were collected as part of the routine diagnostic component of the National Health Laboratory Service (NHLS), after informed consent has been obtained from the patients. To conduct our research study, ethical clearance was obtained from the Human Research Ethics Committee (HREC) of the University of Witwatersrand. Our ethical clearance certificate was assigned the protocol number M220614, Ref number R14/49 and was approved by the ethics committee on 5 July 2022. The 45 TB-HIV human lymph node tissue samples were collected from the Charlotte Maxeke Johannesburg Academic Hospital, Republic of South Africa. These were retrieved from patients who had been on treatment for less than six months. The 45 control tissues were collected from the same hospital and were obtained from individuals without TB-HIV. Specifically, the control samples were obtained from individuals without TB-HIV co-infection and with no clinical history of cancer, high blood pressure, diabetes, allergies, or any other major underlying clinical conditions from the Charlotte Maxeke Johannesburg Academic Hospital. The selected sample size of 90 comprised both patients with TB-HIV (n = 45) and non-TB-HIV patients (n = 45) and was intended to enhance data reproducibility and reliability.

### 2.2. Total RNA Extraction

Total RNA was extracted from 45 infected and 45 control lymph node biopsies using PureLink FFPE Total RNA Isolation Kit (Thermo Fisher Scientific, Waltham, MA, USA) following the manufacture’s recommendations. Reverse transcription is a critical step as it enables conversion of RNA into complementary DNA (cDNA). This conveniently allows amplification and quantification of cDNA by polymerase chain reaction (PCR).

### 2.3. Reverse Transcription

Reverse transcription was accomplished by converting 1 µg of extracted RNA into complementary DNA (cDNA) by utilizing the RevertAid first strand cDNA synthesis kit (Thermo Fisher Scientific, MA, USA) according to the manufacture’s guidelines.

### 2.4. Target Gene Expression by Reverse Transcription Quantitative Polymerase Chain Reaction (RT-qPCR)

The RT-qPCR test was employed to assess gene expression profiling in infected and control tissues. The reaction cocktail comprised 2× iTaq Universal SYBR Green Supermix (Bio-Rad, Hercules, CA, USA), 10 pmol of each primer (Integrated DNA Technologies, Coralville, IA, USA) and 2 µL cDNA (100 ng/µL). Primer sequences used for RT-qPCR experiments are depicted in Supplementary Table S1. Thermal cycling conditions were programmed in the Light Cycler^®^ 480 II (Roche Diagnostics, Mannheim, Germany) as follows: hot start at 95 °C for 30 s, followed by 40 cycles of denaturation at 95 °C for 15 s, annealing at 53 °C for 30 s and extension at 72 °C for 30 s. The *Glyceraldehyde 3-phosphate dehydrogenase* (*GAPDH*) reference gene was included as a positive control. The level of expression for each gene was calculated using the formula ΔCq = 2^Cq target gene-Cq reference gene^, where the quantitation cycle (Cq) represents the average Cq value of target gene minus the mean of *GAPDH* reference gene. Fold change in gene expression was calculated by relativizing the mean 2^Cq target gene^ in infected patients to the average 2^Cq target gene^ in control individuals.

### 2.5. Protein Isolation

Proteins were isolated from lymph node specimens using the Qproteome FFPE Tissue Kit (Qiagen, GmbH, Hilden, Germany) following the manufacturer’s instructions. Protein purity was assessed by measuring absorbance at 280 nm with a NanoDrop.

### 2.6. Determination of Protein Modification

A histone H3 modification multiplex assay kit (LifeSpan Biosciences, Seattle, WA, USA) was employed to determine the degree of histone methylation, acetylation and phosphorylation in proteins isolated from the two study cohorts. The protocol was conducted by following the manufacture’s procedure.

### 2.7. Conventional PCR, DNA Sequencing and Phylogenetic Analysis

Conventional PCR was utilized to amplify cDNA from 10 infected samples. The reaction mixture consisted of 2× PCR master mix (Thermo Fisher Scientific, MA, USA), 10 pmol of each primer pair specific to the HIV *gag-pol* or *Mtb rpoB* gene, and 2 µL cDNA (100 ng/µL). Primer sequences targeting the *gag-pol* and *rpoB* genes are illustrated in Supplementary Table S2. Non-template samples were used as negative controls. The Proflex thermal cycler (Thermo Fisher Scientific, MA, USA) was used and the cycling conditions for amplification were one cycle of initial denaturation at 95 °C for 3 min, 35 cycles of denaturation at 95 °C for 30 s, annealing at 51 °C for 30 s (*rpoB*) or 53 °C for 30 s (*gag-pol*), extension at 72 °C for 1 min. The final extension step was performed at 72 °C for 10 min. Amplicons were electrophoresed on a 2% agarose gel stained with ethidium bromide at a voltage of 100 for 45 min and visualized using the Gel Doc System (Syngene G: Box, Cambridge, UK). Ten randomly selected TB-HIV PCR amplicons were sent to Inqaba (Inqaba, Biotech Industries, Pretoria, South Africa) for sequencing. Nucleotide sequences were compared with GenBank reference sequences deposited in the National Center for Biotechnology Information database using the Basic Local Alignment Search Tool. Our sequences were aligned with GenBank sequences using the multiple sequence alignment tool (DNAMAN version 4.03). The DNAMAN program (DNAMAN version 4.03) was also employed to construct phylogenetic trees using the neighbor-joining method with a bootstrapping stringency of 1000.

### 2.8. Statistical Analysis

Data analysis was achieved by comparing results obtained from 45 control cases and 45 patients co-infected with TB-HIV. Gene expression and protein data were analyzed using the GraphPad Prism software version 7 (GraphPad Software Inc, La Jolla, CA, USA) by applying the non-parametric student *t*-tests. Data were considered statistically significant when *p* < 0.05.

## 3. Results

### 3.1. Variable Target Gene Expression

**Figure 1:**
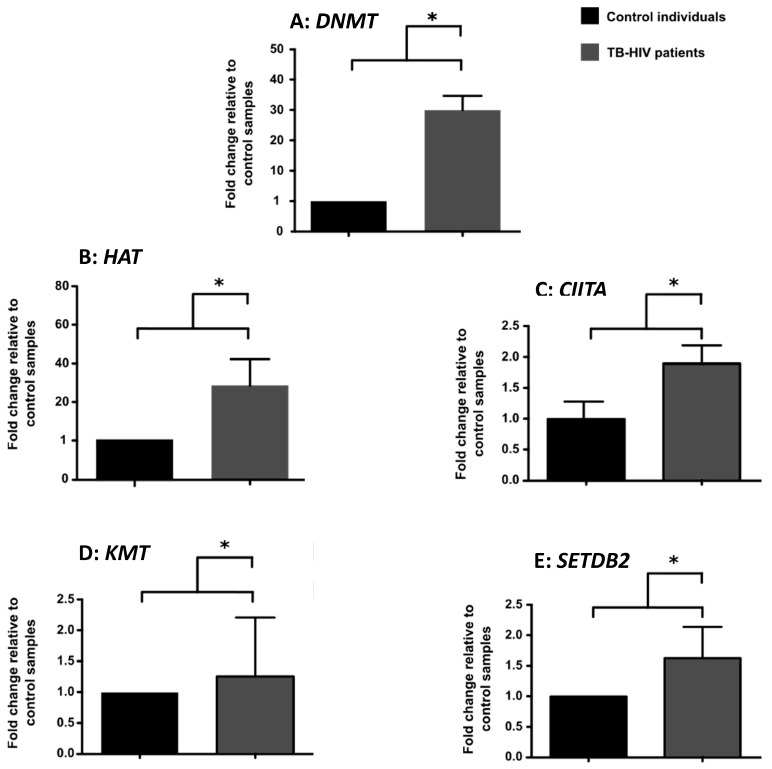
**Gene expression in infected and control individuals.** Target gene amplification was accomplished by RT-qPCR using primers specific for *DNMT1* (**A**); *HAT* (**B**); *CIITA* (**C**), *KMT* (**D**) and *SETDB2* (**E**). Data were obtained from 90 samples representing control individuals (n = 45) and patients co-infected with TB-HIV (n = 45). Values depict the means ±SEM and statistical analysis was conducted using the non-parametric *t*-test. The *p* < 0.05 value (*) indicates statistical significance between the control and experimental samples.

Relative to the controls, exceptional increases in the expression of the *DNMT, HAT, KMT, CIITA* and *SETDB2* genes were observed in the TB-HIV cohort (Figure 1A–E). Importantly, the fold change in gene expression between the two cohorts was statistically significant (Figure 1A–E).

### 3.2. Differential Protein Modification

**Figure 2:**
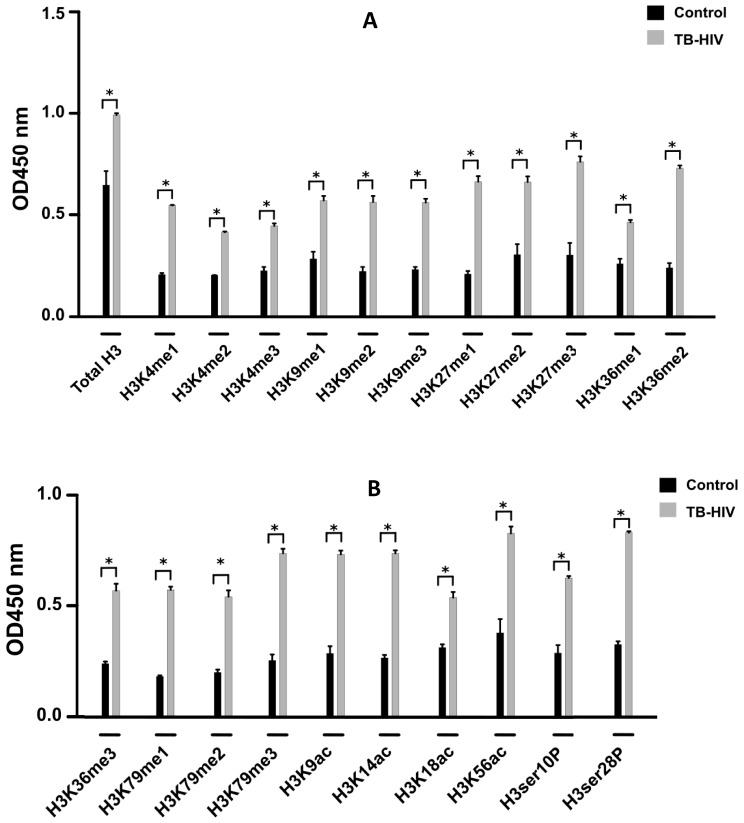
**Degree of histone modification in infected and control individuals.** The level of histone acetylation, methylation and phosphorylation in both study cohorts was determined by the histone H3 modification multiplex assay (**A**,**B**). Data were collected from the control subjects (n = 45) and experimental group (n = 45) and values denote the means ± SEM. Statistical variation between the study cohorts is illustrated by *p* < 0.05 (*).

The histone multiplex assay was used to detect 21 different histone modification patterns in histone H3. Our data revealed that the histone H3 modifications were significantly abundant in the TB-HIV cohort as compared to the control group (Figure 2). Specifically, these epigenetic modifications included methylation, acetylation and phosphorylation (Figure 2).

### 3.3. Convectional PCR and DNA Sequence Analysis

**Figure 3:**
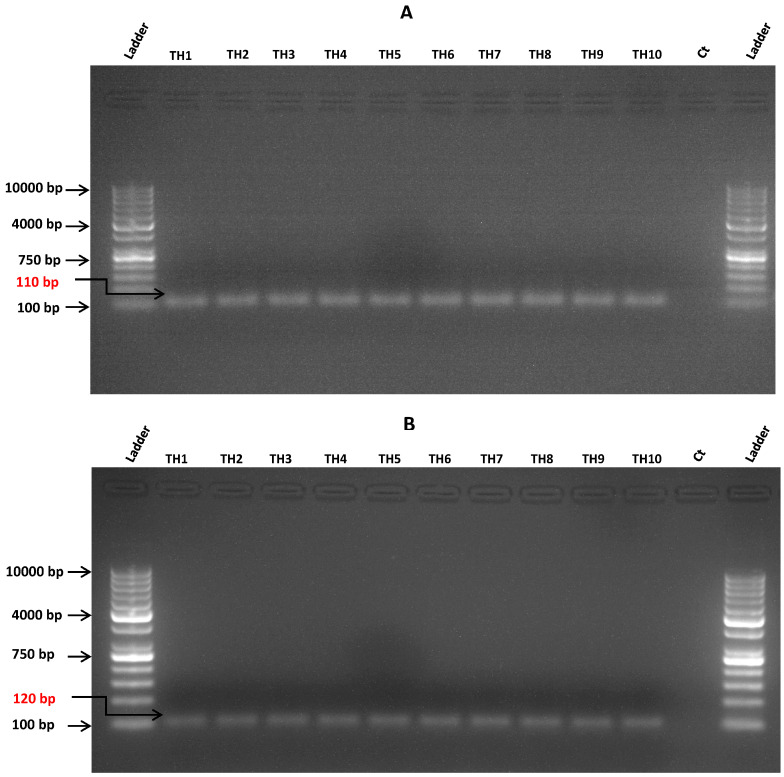
**Amplification of HIV *gag-pol* and *Mtb rpoB* gene by conventional PCR.** The cDNAs were amplified by conventional PCR using primers specific for the HIV *gag-pol* (**A**) and *Mtb rpoB* gene (**B**). Agarose gel electrophoresis revealed the expected PCR product size of about 110 bp (**A**) and 120 bp (**B**) for HIV *gag-pol* and *Mtb rpoB* gene, respectively. A 100 bp DNA ladder was used to determine the size of PCR product. The negative control sample is denoted as Ct.

Conventional PCR confirmed TB-HIV co-infection in 10 randomly selected samples after amplifying the HIV *gag-pol* (Figure 3A) and the *Mtb rpoB* gene (Figure 3B). The DNA fragment size corresponding to HIV *gag-pol* was about 110 bp (Figure 3A), while the band size resulting from *Mtb rpoB* gene amplification was 120 bp (Figure 3B). No amplification was observed in the negative controls (Figure 3A,B).

**Figure 4:**
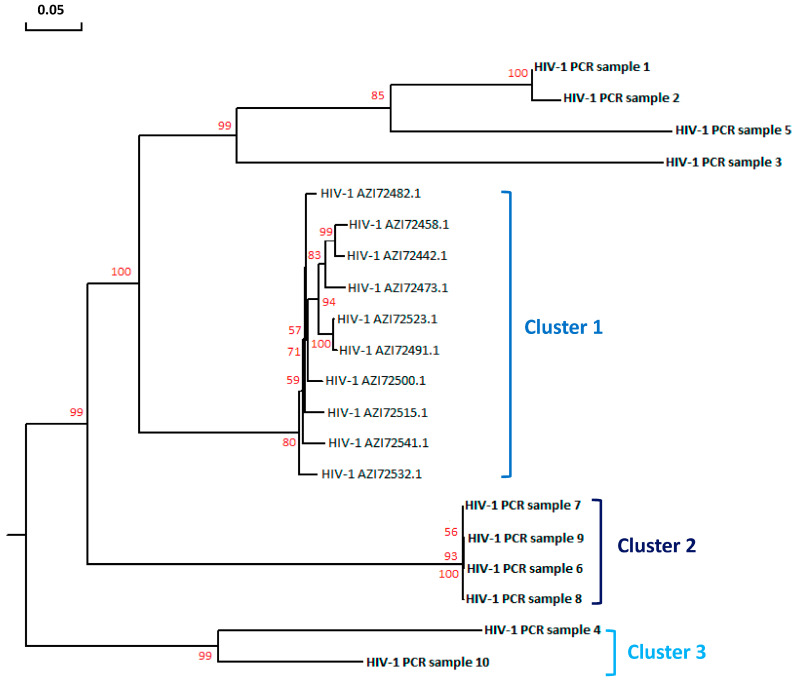
**Phylogenetic analysis of the HIV nucleotide sequences.** The HIV *gag-pol* nucleotide sequences were analyzed by constructing a rooted neighbor-joining tree. The nucleotide sequences from our experimental cohort are shown in bold font (without accession numbers). The accession numbers of related GenBank reference sequences are highlighted in regular font. Branch lengths are proportional to the genetic distance and are denoted by the bar (0.05). Bootstrap values (percentages of 1000 replications) are indicated at the internodes.

**Figure 5:**
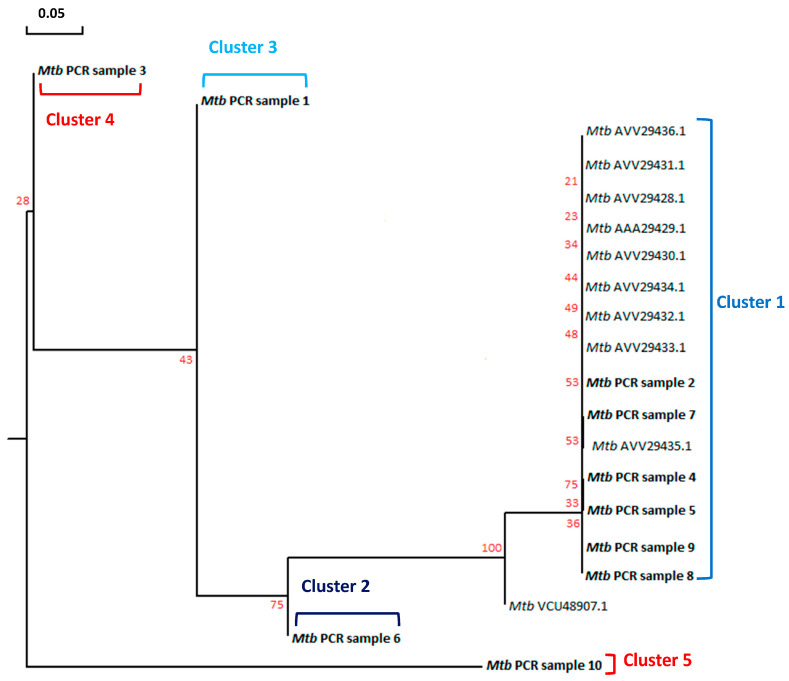
**Phylogenetic analysis of the *Mtb* nucleotide sequences.** The *Mtb rpoB* nucleotide sequences were analyzed by constructing a rooted neighbor-joining tree. The nucleotide sequences from our experimental cohort are shown in bold font (without accession numbers). The accession numbers of related GenBank reference sequences are shown in regular font. The branch lengths are proportional to genetic distance and are indicated by the bar (0.05). Bootstrap values representing 1000 replications are indicated at the internodes.

Phylogenetic analysis of the HIV *gag-pol* sequences revealed that all 10 strains belonged to the HIV-1 subtype C. These isolates were further divided into three distinct clusters (Figure 4). Cluster 1 contained samples 1, 2, 3 and 5 which clustered together with the 10 sequences retrieved from Genbank. A variable phylogenetic profile was observed upon comparing the *rpoB* gene sequences of *Mtb* from the same TB-HIV samples. Five different clusters were observed, with the largest cluster consisting of samples 2, 4, 5, 7, 8 and 9 as well as the nine *Mtb* reference sequences retrieved from the Genbank (Figure 5).

## 4. Discussion

The findings of this study provide valuable insights into the DNA methylation patterns, consistent with previous investigations that demonstrated the correlation between a significant up-regulation of the *DNMT* gene (Figure 1A) which catalyzes methylation and infection with HIV-1 [15,16]. Furthermore, our findings validate previous investigations that highlighted the effect of significant DNA methylation leading to elevated expression of *interleukin-17* gene which compromises the host immune response [17,18]. Nevertheless, our DNA methylation results (Figure 1A) were inconsistent with the data reported by Marimani et al., 2020, who observed a lower methylation profile in the TB-HIV co-infected cohort relative to the healthy subjects [19]. SETDB2 is a protein that regulates gene expression by catalyzing methylation at Histone3 (H3) and Lysine 9 (K9), and its function is particularly involved in transcriptional silencing [20]. Therefore, the elevated expression of the *SETDB2* gene indicated by our results (Figure 1E), as well as the increased H3K9 methylation (Figure 2A), suggests that, during co-infection with TB-HIV, there is significant transcriptional silencing. These findings corroborate a study by Maricato et al., 2015, which also demonstrated that infected blood cells exhibit increased expression of the *SETDB2* gene [20].

The KMT protein is an important regulator of the methylation of various lysine residues. Our findings suggest that the up-regulation of *KMT* gene expression (Figure 1D) in patients co-infected with TB-HIV is associated with the viral replication. The study by Pegans et al., 2010 [21], demonstrated that lysine methyltransferase enhances HIV transcription by monomethylating the viral transactivator Tat protein. This observation supports our current findings and indicates that the inhibition of *KMT* activity could be a potential method of suppressing viral replication [22]. The histone H3 methylation profile was sharply increased in the patients with TB-HIV (Figure 2) and confirmed the gene expression data (Figure 1D).

The signature histone acetylation patterns detected by the histone H3 assay included H3K9, H3K14, H3K18 and H3K56 (Figure 2B) which are typically associated with gene activation. Gene activation generally increases access to the chromatin and leads to significant expression of the target genes [23]. Our results demonstrated that the degree of histone H3 acetylation was markedly higher in individuals co-infected with TB-HIV (Figure 2A,B). Our findings were also consistent with a prior study that reported elevated expression of *HAT* in patients positive for HIV-1 following *Mtb* infection [24]. The *CIITA* gene involved in histone acetylation was also markedly increased in the TB-HIV co-infected cohort (Figure 1C). Notably, these findings (Figure 1C) align with an earlier investigation conducted by Porter et al., 2010 [25,26].

A considerable increase in the level of histone H3 phosphorylation at serine residues 10 and 28 was observed in patients diagnosed with TB-HIV (Figure 2B). Phosphorylation at serine 10 and 28 plays a crucial role in activating host immunity against a wide variety of stimuli [27,28]. Our results also confirm that phosphorylation is induced in response to TB-HIV co-infection (Figure 2B). This is also in agreement with prior investigations that demonstrated a protective phosphorylation profile following infection with HIV or TB [29,30]. Overall, a considerable increase in histone H3 acetylation, methylation and phosphorylation (Figure 2A,B) was induced by a marked transcriptional upregulation of genes associated with acetylation, methylation and phosphorylation, respectively (Figure 1A–E). This observation may suggest that, following microbial infection, *Mtb* and HIV dysregulate the normal epigenetic mechanisms such as acetylation, methylation and phosphorylation leading to aberrant increases in target gene transcription. This upregulation in target gene expression ultimately leads to production of aberrant histone H3 proteins with enhanced acetylation, methylation and phosphorylation levels.

The agarose gel electrophoresis results (Figure 3A,B) indicated that the target genes were successfully amplified by conventional PCR in the HIV and TB co-infected tissue samples. This resulted in amplicon band sizes of approximately 110 bp for the HIV *gag-pol* (Figure 3A) gene and 120 bp for the *Mtb rpoB* gene (Figure 3B). The phylogenetic analysis revealed that all the HIV isolates belong to the HIV-1 subtype C strain (Figure 4). Variations in the relationships among the HIV strains highlight genetic divergence that potentially resulted from mutations within the *gag-pol* gene in response to treatment with HIV protease inhibitors. The phylogenetic profile of the *Mtb rpoB* sequences from the same cohort revealed five different clusters (Figure 5). The genetic divergence among the various strains emanated from genetic mutations within the *rpoB* gene. These mutations are likely to have occurred due to the high selection pressure exerted on the anti-TB drug rifampicin which is used for routine TB treatment. Limitations of the current study include the use of biopsy specimens recovered from patients without TB-HIV co-infection and the lack of whole genome methylation, acetylation and phosphorylation data. Therefore, subsequent studies will aim to specifically unravel the microbial contribution to the regulation of epigenetic mechanisms in patients with HIV that do not have TB (group 1), in patients with TB without HIV (group 2) and in healthy individuals without TB-HIV co-infection (group 3). This study design will allow for the specific detection of the degree of the epigenetic mechanism that is regulated by HIV or *Mtb* following human infection. Importantly, patients and healthy individuals from different racial groups, genders, ethnicities and geographical locations will be recruited to partake in this prospective investigation. This practice will yield comprehensive data on the impact of gender, race, ethnicity and environment on microbial infection, disease progression, immune response and the degree of epigenetic dysregulation. Whole genome sequencing data will enable identification of additional gene regions associated with acetylation, methylation and phosphorylation. Accurate identification of these target genes will provide a better understanding of the molecular mechanisms responsible for gene dysregulation, aberrant protein production levels and the role of these candidate genes in modulating the host’s immune response.

## 5. Conclusions

The key genes explored in this study may lead to a better understanding of the epigenetic marks that occur in the presence of TB-HIV infection and could potentially function as biomarkers for the development of novel diagnostic tools as well as anti-HIV and anti-TB treatments.

## Data Availability

Data are contained within the article.

## Supplementary Materials

The following supporting information can be downloaded at: https://www.mdpi.com/article/10.3390/microorganisms12051001/s1, Table S1: Primer sequences of candidate genes and *GAPDH* reference gene used for RT-qPCR; Table S2: Primer sequences for HIV *gag-pol* and *Mtb rpoB* genes used for conventional PCR.
