# Supplementary material for: Differential Epigenetic Regulation in Uninfected and Tuberculosis–Human Immunodeficiency Virus Co-Infected Patients
Source: Microorganisms. 2024 May 16;12(5):1001. doi: 10.3390/microorganisms12051001 (PMC11123988; doi:10.3390/microorganisms12051001)
Supplement: Supplementary file 1 [file microorganisms-12-01001-s001.zip › microorganisms-2992843-supplementary.pdf]

Supplementary Table S1. Primer sequences of candidate genes and *GAPDH* reference gene used for RT-qPCR

Primer name	Orientation	Primer sequence	PCR DNA fragment size
Human CIITA F<br>Human CIITA R	Forward<br>Reverse	5’-AGAAGTCCAAGGTTAGGGGC-3’<br>5’-GGGTTGTCGTGGGAGTAAGA-3’	164 bp
Human HAT F<br>Human HAT R	Forward<br>Reverse	5’-CCCGTGGTGACTGTTTTTCT-3’<br>5’-ATGCCCTTACCTCACACCTG-3’	174 bp
Human DNMT1 F<br>Human DNMT1 R	Forward<br>Reverse	5’-GTGGGGGACTGTGTCTCTGT-3’<br>5’-AGGAGCACTCGTCCTTTTCA-3’	185 bp
Human SETDB2 F<br>Human SETDB2 R	Forward<br>Reverse	5’-AAATGACTGGCTTGCCAAGG-3’<br>5’-TAACACACTGCTCGGGAAGT-3’	202 bp
Human KMT F<br>Human KMT R	Forward<br>Reverse	5`-TCAACCTGCTCTCCTTCCAG-3`<br>5`-AAGGGAAGAACAGGGATGGG-3`	230 bp
Human GAPDH F<br>Human GAPDH R	Forward<br>Reverse	5’-CCCTAGTCCCCAGAAACAGG-3’<br>5’-ATTTCCTTCCCGGTTGCAAC-3’	232 bp

Supplementary Table S2. Primer sequences of HIV *gag-pol* and *Mtb rpoB* genes used for conventional PCR

Primer name	Orientation	Primer Sequence	PCR DNA fragment size
HIV gag-pol F<br>HIV gag-pol R	Forward<br>Reverse	5’-CCAGCGGCTACACTAGAAGA-3’<br>5’-CCTGCAATTTCTGGCTGTGT-3’	110 bp
Mtb rpoB F<br>Mtb rpoB R	Forward<br>Reverse	5’-GTCGACGAGTGCAAAGACAA-3’<br>5’-TGGTCTCGTCGAAGTACACC-3’	120 bp
